# Latent Profile Analysis of Mental Health Among Children and Young Adults With Refugee Backgrounds

**DOI:** 10.1016/j.jaacop.2025.06.003

**Published:** 2025-06-16

**Authors:** Johan Andersson, Hongru Zhai, Reeta Kankaanpää, Carolina Bråhn, Erica Mattelin, Kirsi Peltonen, Ann-Charlotte Münger, Laura Korhonen

**Affiliations:** a Linköping University, Linköping, Sweden; b University of Turku, Turku, Finland; c Save the Children, Stockholm, Sweden

**Keywords:** refugees, child, young adult, mental health, latent variable modeling

## Abstract

**Objective:**

Children and young adults comprise a significant proportion of the world’s refugee population and are disproportionately negatively affected by the social determinants of health. This heterogeneous group faces high rates of poor mental health, yet research investigating within-group inequalities in mental health remains limited. We performed a latent profile analysis to explore classes of mental health based on posttraumatic stress symptoms (PTSS), general functioning, and well-being. This study aimed to improve the understanding of mental health differences, thereby providing better guidance for assessment and tailored interventions.

**Method:**

This study involved 131 children and 127 young adults with refugee backgrounds (mean age 18.21 years, 44.6% female, 23.6% unaccompanied) recruited nationwide in Sweden (2019-2022). To examine classes and their predictors, latent profile analysis was conducted, followed by multinomial logistic regression analysis.

**Results:**

Latent profile analysis identified four distinct classes: Good Mental Health (58.1%; low PTSS, good functioning and well-being); Severe Mental Distress (13.6%; high PTSS, low functioning and well-being); Moderate Mental Strain (12.4%; low PTSS, moderate functioning, low well-being); and Resilient (15.9%, high PTSS, good functioning, moderate well-being). Social determinants of health, such as being unaccompanied, asylum status, exposure to multiple types of violence, sexual victimization, and child maltreatment, distinguished the classes.

**Conclusion:**

Children and young adults with refugee backgrounds can be categorized into classes based on clinically relevant mental health indicators. Focusing solely on those individuals at the highest risk for poor mental health may overlook many who are mentally healthy and those who need more targeted support. Future research should aim to replicate findings and to evaluate additional predictive factors at the family and societal levels.

## Plain language summary

This study found that a Swedish community sample of children and young adults with refugee backgrounds could be grouped into four mental health profiles: good mental health, resilient, moderate mental strain, and severe mental distress. While most participants were doing well or coping despite challenges, others showed signs of moderate or severe mental health difficulties. Exposure to violence, being unaccompanied, and insecure asylum status were linked to poorer mental health. Focusing only on those in obvious distress may overlook both those in need of more targeted support and those who are doing well.

Mental health is a state of emotional, psychological, and social well-being that enables individuals to cope with stressors, to relate to others, and to function well in daily life.^1^ When understood this way, good mental health extends beyond the mere absence of symptoms: it also encompasses positive well-being and the ability to perform well in various aspects of life.^2^ The dual-factor model of mental health recognizes this, stating that mental health consists of 2 separate but intercorrelated domains: mental well-being and mental illness.^3^ Evidence supporting the dual-factor model is growing, emphasizing the importance of considering both dimensions when measuring mental health and implementing interventions.^4^ A related, yet separate concept from mental health, is resilience. Fundamentally defined as better-than-expected outcomes despite the experience of adversity,^5^ resilience has been described as the ability to use resources to overcome challenges^6^ and as the capacity of a dynamic system to adapt to significant threats.^7^

Social factors such as unmet basic needs, poverty, limited access to education, and adverse life experiences such as violence influence mental health throughout a person's life. These factors are known as social determinants of health.^8^ These circumstances do not have an equal impact on individuals; instead, inequality is the primary driving force behind these social determinants, whether comparing groups within countries and regions or at a global level.^9^

Children and young adults with refugee backgrounds, comprising over 40% of the more than 117 million people forcibly displaced worldwide,^10^ are a group disproportionately affected by social determinants of mental health. Consequently, this group is frequently labeled as vulnerable and needing support.^11^ Previous studies have supported this view, showing higher rates of exposure to violence as well as posttraumatic stress disorder (PTSD), depression, and anxiety disorders compared to general populations.^12^, ^13^, ^14^ Simultaneously, Gadermann *et al.*^15^ showed that children with immigrant or refugee backgrounds typically exhibited lower diagnostic prevalence rates of mental disorders, and a recent Swedish study^16^ estimated relatively low prevalence rates for mental disorders, along with favorable self-reported well-being and clinician-rated general functioning. In a similar vein, researchers have started to highlight the importance of shifting focus from vulnerability (ie, illness) to strength by concentrating more on resilience when studying populations with refugee backgrounds, aiming to enhance interventions designed to improve mental health among this group.^17^^,^^18^

Methodological differences can partially explain the high variability in prevalence rates of mental health disorders, but substantial population heterogeneity also exists. Individuals with refugee backgrounds come from many different countries and cultures. They are forced to flee for a variety of reasons, including war, natural disasters, and violations of human rights. Some have experienced rather ordinary childhoods, whereas others have spent their entire lives in war zones or refugee camps, and the circumstances in the destination countries vary significantly.^19^^,^^20^ In addition, the stigma (defined as mark of disgrace, shame, and disapproval resulting from discrimination, rejection, and exclusion of a person from various areas of society^21^ and including self-stigma, public stigma, professional stigma, and institutional stigma) surrounding mental health in populations with refugee backgrounds may also contribute to the variability in prevalence rates.^22^

Given this heterogeneity, studies on within-group inequalities in mental health are needed. An increased understanding of this diversity could improve assessments and assist in developing more tailored interventions. Recently, there has been a growing interest in using person-centered statistical analyses, such as latent profile analysis (LPA),^23^ to disentangle mental health differences.^24^ However, these methods have only been applied to a limited extent to study children and young adults with refugee backgrounds.^25^

### Aim and Hypotheses

This study aimed to examine heterogeneity in mental health in a Swedish sample of children and young adults with refugee backgrounds. Our hypotheses were as follows: (1) Meaningful classes based on mental health indicators would be identified. (2) The classes would be characterized by varying profiles of posttraumatic stress symptoms (PTSS), general functioning, and well-being. (3) At least one highly affected class and a thriving class would be identified. (4) Combinations of covariates could predict class membership.

## Method

### Study Setting and Sample

This study was part of a larger research project entitled “Long Journey to Shelter,” which aims to understand how various factors affect refugee mental health and integration into Swedish society.^26^ The study has collected data from adolescents and adults with refugee backgrounds (ie, refugees, asylum seekers, family reunification migrants, quota refugees, and undocumented migrants) 12 to 25 years of age through structured and semi-structured interviews, along with saliva and hair sampling. Measures include sociodemographic data and data on mental health and its determinants, including genotypes, stress hormone levels, access to healthcare, and the migration process, including settlement in Sweden. The sample consists primarily of participants from the Middle East, North Africa, and sub-Saharan Africa. Nearly the entire study sample had experienced violence, but the prevalence rates for all psychiatric diagnoses in the whole sample were lower than those reported in earlier published studies. There were no significant differences in diagnosis prevalence by gender. Self-reported well-being and observer-rated functional ability were high when assessing the overall study population. However, being an unaccompanied refugee was associated with a higher risk of suicidal thoughts, higher rates of PTSS, lower mental well-being, and lower functional ability.^16^

The study and its amendments received approval from the Regional Ethics Board of Linköping (2018/292-31 and 2018/504-32) and the National Ethics Board (2019-05473, 2020-00949, and 2021-03001).

The original sample consisted of 2 groups: (1) children and adolescents 12 to 17 years of age who had applied for asylum in Sweden no more than 6 months before assessment (n = 161); and (2) young adults 18 to 25 years of age with no time limitation on their stay in Sweden since their asylum application (n = 135). A small number of participants in the first group turned 18 years of age between agreeing to participate in the study and the formal interview. They were still included in the sample but were only counted in the first group. All participants had a refugee background. Participants who did not complete the trauma questionnaires were excluded from this study, resulting in a final sample size of 258.

Young adults up to age 25 years were included to encompass the entire period of adolescence, following the contemporary definition of this period of life as spanning 10 to 24 years.^27^ By expanding the traditional age range associated with adolescence, this study addresses the crucial biological and social development during this time, which is essential for mental health development.

### Procedure

Data were collected through structured quantitative interviews between 2019 and 2022. Convenience sampling was used. Participants were recruited through social services, schools, health care, social media, and nongovernmental organizations. Professionals (eg, teachers, social workers, and health care professionals) working with the target group within these organizations volunteered to assist recruitment by asking potential participants or their legal guardians if they wanted to participate in the study. If interested, they received a leaflet and were contacted by one of the researchers via phone. All study information was provided verbally and in writing in the participant’s preferred language. Written consent was obtained from all participants. The assessment, including demographic information and questionnaires, was conducted through structured quantitative interviews in the participants’ preferred language. Most interviews used an interpreter, whereas some were conducted in English. An Arabic-speaking research assistant interviewed most Arabic-speaking participants. Some interviews took place in person at a location chosen by the participant, whereas others had to be conducted online via Zoom because of COVID-19 pandemic restrictions. More information about the procedure can be found in the published protocol^26^ and the primary study of the larger research project.^16^

### Covariates

The following information was gathered through direct questioning during the interviews: age, gender (male/female/other), country of origin, asylum status (awaiting decision/decision received/appealing/do not know), years in Sweden, parental education for both parents, unaccompanied status, and whether participants had ever sought care for a mental health issue. Details about the transformation of variables for regression analysis can be found in Supplement 1, available online. Many study participants expressed uncertainty regarding their race and ethnicity, which is why data are not reported.

### Measures

The study measured exposure to violence using the Juvenile Victimization Questionnaire (JVQ), a 34-item questionnaire designed to capture a wide range of victimization experiences.^28^ The questionnaire was adapted for this study by including experiences related to natural disasters, accidents, medical procedures, death of close relatives, poverty, separation from their parents, being captured or imprisoned, and human trafficking. PTSS in children were assessed using the Child and Adolescent Trauma Screen version 1 (CATS-1), a 20-item scale based on the *DSM-5* criteria for PTSD.^29^ For young adults, the 20-item Posttraumatic Stress Disorder Checklist for *DSM-5* (PCL-5) was used.^30^ Mental well-being was assessed through the 5-item World Health Organization Well-Being Index (WHO-5).^31^ General functioning was measured with the clinician-rated Global Assessment of Functioning (GAF)^32^ and the Children’s Global Assessment Scale (CGAS).^33^ Resilience was assessed using 5 items from the Adolescent Resilience Questionnaire (ARQ).^34^ To determine how many participants met the diagnostic criteria for common mental disorders, the Mini International Neuropsychiatric Interview for Children and Adolescents 6.0 (MINI-KID)^35^ was administered for children, whereas the Mini International Neuropsychiatric Interview 7.0 (MINI)^36^ was used for young adults. Internal consistency for CATS-1 (α = 0.94), PCL-5 (α = 0.90), and WHO-5 (α = 0.86) were strong in the sample. Additional details on the measures can be found in Supplement 1, available online.

### Data analysis

LPA was usd to examine latent mental health classes, and the reporting follows the adapted Guidelines for Reporting on Latent Trajectory Studies (GRoLTS) checklist.^24^ CATS-1/PCL-5, GAF/CGAS, and WHO-5 were used as indicators for the classes to capture the different dimensions (symptoms, general functioning, and well-being) of mental health. PTSS was chosen as the symptom indicator because of the high prevalence of these symptoms in previous studies on people with refugee backgrounds^12^^,^^13^ and because it was the only continuous symptom variable available in the data.

Scores on all indicators were standardized for ease of comparison. LPA was run in R using the tidyLPA package.^37^ Missing data on individual items in CATS and PCL-5 were handled with mean imputation using available item data within each questionnaire. This approach was chosen because missingness was minimal (0.5% in CATS and 0.4% in PCL-5), and leveraging existing questionnaire responses allowed for the most accurate estimation of missing values. In contrast, missing data on total scores for WHO-5 and GAF were more substantial (3.5% and 1.6%, respectively), and for the WHO-5, entire questionnaires were often missing rather than individual items. To maximize available data in these instances, we applied the MissForest random forest imputation algorithm, which uses patterns across all available variables to provide more robust estimates.^38^ For the covariates used in the regression analysis, the percentage of missing data ranged from 0% to 8.1%, and analysis was run with complete case data.

To determine the optimal number of groups, the Bayesian information criterion (BIC)^39^ and the Akaike information criterion (AIC)^40^ were examined for model selection along with significance on the bootstrapped likelihood ratio test (BLRT).^41^ High entropy was sought, along with sensible class sizes. Six different models with varying variance and covariance restrictions were fitted. In all models, we tested 1 to k-classes, stopping when fit indices indicated that adding more classes was related to worse fit. Supplement 1, available online, provides further details on model parameters and model selection.

We conducted a multinomial logistic regression analysis to examine significant predictors of class membership. We first ran an unadjusted model and then an adjusted model, including confounders. Minimal sufficient adjustment sets were identified through directed acyclic graphs based on theoretical associations.^42^ Because the sample included 2 groups (ie, children and young adults), additional analyses were conducted using age as a continuous variable and categorical variable (12-14, 15-17, 18-21, and 22-25). Group (child/adult) was also included as a variable to assess potential group effects. When confounders were included in the model, the results remained consistent across these approaches. Therefore, age was treated as a continuous variable in subsequent analyses. Details of these analyses are presented in Tables S6 and S7, both available online.

Regression analysis was performed in R using the nnet package.^43^ Because of the small sample size, we could not test the reliability of classes through split samples.

## Results

### Sample Characteristics

Table 1 summarizes the sociodemographic information of the participants. The mean age of the total sample was 18.21 years, 44.6% of all participants were female, and 23.6% arrived in Sweden unaccompanied.

**Table 1:** Sociodemographic Characteristics of the Sample

Variable	Total (N = 258)	Children (n = 131)	Young adults (n = 127)
Age, y, mean (SD)	18.21 (3.75)	15.15 (1.83)	21.38 (2.31)
Minimum/maximum	12/25	12/18	18/25
Missing	0	0	0
Gender, female, n (%)	115 (44.6)	58 (44.3)	57 (44.9)
Missing	0	0	0
Region of origin, n (%):			
Middle East and North Africa	189 (73.5)	66 (50.4)	123 (97.6)
Sub-Saharan Africa	61 (23.7)	58 (44.3)	3 (2.4)
Other	7 (2.7)	7 (5.3)	0
Missing	1		1
Asylum status, n (%):			
Decision received	200 (77.5)	79 (60.3)	121 (95.3)
Awaiting decision	36 (14.0)	31 (23.7)	5 (3.9)
Appealing	4 (1.6)	3 (2.3)	1 (0.8)
Do not know	18 (7.0)	18 (13.7)	0
Missing	0	0	0
Years in Sweden, mean (SD)	2.71 (2.95)	0.30 (0.14)	5.21 (2.31)
Minimum/maximum	0.08/13.42	0.08/1	0.08/13.42
Missing, n	2	1	1
Unaccompanied, n (%)	61 (23.6)	19 (14.5)	42 (33.1)
Missing	0	0	0
Parental education, (%)			
High	94 (38.1)	21 (16.8)	73 (59.8)
Medium	49 (19.8)	24 (19.2)	25 (20.5)
Low	104 (42.1)	80 (64.0)	24 (19.7)
Missing	11	6	5

### Latent Profile Analysis

**Figure 1:**
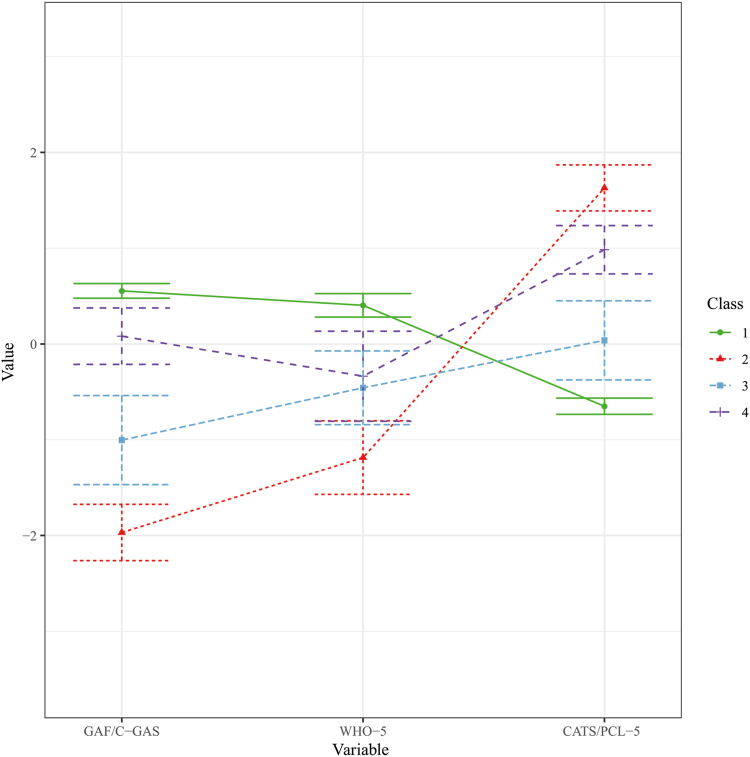
Profile Plot for Model 1 With 4 Classes ***Note:****Values standardized for ease of comparison.*

Initial analysis showed the most promising solutions to be model 1 with 4 classes (1:4), model 2 with 3 classes (2:3), and model 6 with 3 classes (6:3). Stability was achieved with all 3 solutions. In all solutions, the smallest classes comprised >10% of the total sample, deemed acceptable.^44^ Model indices AIC and BIC were inconsistent. Entropy was the highest for model 1:4, with similar numbers for 2:3 and 6:3. Upon further examination of the plots, it was noted that 1:4 provided a more distinct delineation between classes and that 2:3 and 6:3 were very similar. Furthermore, the plots for models 2:3 and 6:3 indicated that the classes might merely reflect scales of severity on the measured indicators, known as the salsa effect.^44^ In contrast, model 1:4 not only aligned with our hypothesis of a poor mental health class and a good mental health class, but also yielded theoretically meaningful results in 2 additional classes distinguished by disparate outcomes in functioning and PTSS. Therefore, this was the final selected model. Table S3, available online, presents the fit indices for model 1. The profile plot for model 1:4 can be found in Figure 1. Fit indices and profile plots for the other evaluated models can be found in Supplement 1, available online.

Class 1 was characterized by good general functioning (GAF/CGAS mean 89.39), positive well-being (WHO-5 mean 79.87), and low PTSS (CATS-1 mean 4.09 and PCL-5 mean 17.18). This class was named “Good Mental Health” and comprised 58.1% of the sample. In contrast, class 2 was distinguished by moderate-to-low functional impairment (GAF/CGAS mean 53.47), low well-being with scores above the recommended cut-off (≥50) for depression,^31^ and high PTSS above the recommended screening threshold for PTSD (≥21 on CATS-1^45^ and ≥31-33 on PCL-5^46^). This class was named “Severe Mental Distress” and comprised 13.6% of the sample. Class 3, termed “Moderate Mental Strain,” exhibited moderate functional impairment (GAF/CGAS mean 66.06), moderate well-being (WHO-5 mean 59.00), and moderate-to-low PTSS (CATS-1 mean 11.85 and PCL-5 mean 29.86) and made up 12.4% of the sample. Class 4 displayed good general functioning (GAF/CGAS mean 82.66), moderate well-being (WHO-5 mean 60.68), and high PTSS above screening thresholds, and was thus designated “Resilient” (referring to successful adaptation despite high symptom burden) and consisted of 15.9% of the sample. Descriptive statistics on the class indicators for the total sample and each class are presented in Table 2.

**Table 2:** Descriptives for Class Indicators for Total Sample and Classes

Indicator variable	Total (N = 258)	Class 1 Good mental health (n = 150)	Class 2 Severe mental distress (n = 35)	Class 3 Moderate mental strain (n = 32)	Class 4 Resilient (n = 41)
GAF/C-GAS, mean (SD)	80.65 (14.64)	89.39 (5.76)	53.47 (8.83)	66.06 (7.16)	82.66 (7.11)
Minimum/maximum	31/100	75/100	31/69	50/77	68/96
Missing, n	4	2	1	1	0
WHO-5, mean (SD)	69.56 (24.59)	79.87 (18.30)	41.32 (24.90)	59.00 (23.40)	60.68 (22.40)
Minimum/maximum	0/100	32/100	0/100	20/100	8/100
Missing, n	9	2	4	4	0
CATS-1, mean (SD)	11.77 (13.07)	4.09 (4.72)	37.20 (7.81)	11.85 (5.67)	26.51 (6.04)
Minimum/maximum	0/54	0/16	25.26/54.00	0/20	15/36
Missing, n	0	0	0	0	0
PCL-5, mean (SD)	29.91 (17.09)	17.18 (8.39)	53.32 (9.73)	29.86 (8.26)	46.12 (7.03)
Minimum/maximum	0/69	0/36	33/69	12/43	33.79/62.00
Missing, n	0	0	0	0	0

Note: CATS-1 = Child and Adolescent Trauma Screen version 1; C-GAS = Children’s Global Assessment Scale; GAF = Global Assessment of Functioning; PCL-5 = Posttraumatic Stress Disorder Checklist for *DSM-5*; WHO-5 = World Health Organization Well-being Index.

### Predictors of Class Membership

Table 3 presents descriptive statistics on covariates for the total sample and individual classes, along with the results of the unadjusted multinomial logistic regression analysis. Descriptive statistics on additional covariates can be found in Table S6, available online.

**Table 3:** Descriptives for Covariates for Total Sample and Classes and Differences Between Classes Based on Unadjusted Regression Model

Covariates	Total (N = 258)	Class 1 Good mental health (n = 150)	Class 2 Severe mental distress (n = 35)	Class 3 Moderate mental strain (n = 32)	Class 4 Resilient (n = 41)	Significant differences between classes (*p* < .05)
Age, years, mean (SD)	18.21 (3.75)	17.18 (3.79)	18.83 (3.30)	18.28 (3.66)	19.22 (3.86)	C1 < C4
Minimum/maximum	12/25	12/25	14/25	12/25	12/25	
Missing	0	0	0	0	0	
Gender, female, n (%)	115 (44.6)	71 (47.3)	16 (45.7)	11 (34.40)	17 (41.50)	
Missing	0	0	0	0	0	
Region of origin, n (%):						
Middle East and North Africa	189 (73.5)	109 (72.7)	25 (71.4)	19 (61.3)	36 (87.8)	
Sub-Saharan Africa	61 (23.7)	38 (25.3)	9 (25.7)	10 (32.2)	4 (9.8)	C4 < C1, C3
Other^a^	7 (2.7)	3 (2.0)	1 (2.9)	2 (6.5)	1 (2.4)	
Missing	1	0	0	1	0	
Asylum status, n (%)^b^						
Decision received	200 (77.5)	120 (80.0)	22 (62.9)	24 (75.0)	34 (82.9)	
Awaiting decision	36 (14.0)	15 (10.0)	11 (31.4)	5 (15.6)	5 (12.2)	C2 > C1, C4
Appealing	4 (1.6)	0 (0)	2 (5.7)	1 (3.1)	1 (2.4)	
Do not know^a^	18 (7.0)	15 (10.0)	0 (0)	2 (6.3)	1 (2.4)	
Missing	0	0	0	0	0	
Years in Sweden, mean (SD)	2.71 (2.95)	2.44 (2.87)	3.55 (3.16)	3.05 (3.57)	2.74 (2.39)	C2 > C1
Minimum/maximum	0.08/13.42	0.08/13.42	0.08/9.25	0.08/12.00	0.08/7.67	
Missing	2	2				
Unaccompanied status, n (%)	61 (23.6)	19 (12.7)	19 (54.3)	9 (28.1)	14 (34.1)	C1 < C2, C3, C4
Missing	0	0	0	0	0	C2 > C3, C4
Parental education,^c^ (%)						
High	94 (38.1)	59 (40.7)	11 (34.3)	9 (30.0)	15 (37.5)	C1 > C2
Medium	49 (19.8)	28 (19.3)	5 (15.6)	7 (23.3)	9 (22.5)	C1 < C2
Low	104 (42.1)	58 (40.0)	16 (50.0)	14 (46.7)	16 (40.0)	C1 < C2
Missing	11	5	3	2	1	
ARQ items, mean (SD)						
ARQ Self	3.67 (1.20)	3.69 (1.27)	3.43 (1.16)	3.67 (0.96)	3.87 (1.10)	
Minimum/maximum	1/5	1/5	1/5	1/5	1/5	
Missing	9	5	0	2	2	
ARQ Family	4.10 (1.16)	4.21 (1.09)	3.89 (1.35)	4.07 (1.09)	3.88 (1.29)	
Minimum/maximum	1/5	1/5	1/5	1/5	1/5	
Missing	6	5	0	1	0	
ARQ Peers	4.00 (1.40)	4.23 (1.25)	3.15 (1.58)	4.13 (1.31)	3.81 (1.58)	C1 > C2; C2 < C3
Minimum/maximum	1/5	1/5	1/5	1/5	1/5	
Missing	3	1	1	1	0	
ARQ School	3.03 (1.40)	3.08 (1.49)	3.12 (1.49)	3.13 (1.34)	2.73 (1.60)	
Minimum/maximum	1/5	1/5	1/5	1/5	1/5	
Missing	7	5	1	1	0	
ARQ Society	3.40 (1.36)	3.64 (1.23)	3.02 (1.56)	3.43 (1.36)	2.88 (1.49)	C1 > C2, C4
Minimum/maximum	1/5	1/5	1/5	1/5	1/5	
Missing	9	7	0	2	0	
Exposure to number of different types of violence, mean (SD)	10.97 (7.22)	8.52 (6.32)	17.71 (7.41)	12.31 (7.39)	13.07 (5.33)	C1 < C2, C3, C4
Minimum/maximum	0/34	0/25	4/34	0/25	5/28	C2 > C3, C4
Missing	1	1	0	0	0	
Exposure to sexual victimization, yes, n (%)	66 (25.8)	24 (16.1)	19 (55.9)	7 (21.9)	16 (39.0)	C1 < C2, C4
Missing	2	1	1	0	0	C2 > C3
Exposure to child maltreatment, yes, n (%)	120 (46.9)	55 (36.9)	28 (82.4)	15 (46.9)	22 (53.7)	C1 < C2
Missing	2	1	1	0	0	C2 > C3, C4
Internalizing diagnosis, n (%)	46 (18.3)	6 (4.1)	21 (65.6)	8 (26.7)	11 (26.8)	C1 < C2, C3, C4
Missing	7	2	3	2	0	C2 > C3, C4
Number of diagnoses, mean (SD)	0.57 (1.18)	0.11 (0.39)	2.39 (1.95)	0.77 (1.09)	0.63 (0.86)	C1 < C2, C3, C4
Minimum/maximum	0/7	0/3	0/7	0/4	0/3	C2 > C3, C4
Missing	5	2	2	1	0	
Sought treatment, yes, n (%)	57 (22.7)	18 (12.1)	20 (62.5)	10 (34.5)	9 (22)	C1 < C2, C3
Missing	7	1	3	3	0	C2 > C3, C4

Note: Missing values are reported for individual items, except for exposure to number of different types of violence, exposure to sexual victimization, and exposure to child maltreatment, for which “missing values” indicates the whole scale. ARQ = Adolescent Resilience Questionnaire.

a Not included in analysis of differences between classes.

b Asylum status was dichotomized into 2 categories (awaiting decision/decision received) for analysis of differences between classes.

c High is analyzed with low or medium as reference category; medium is analyzed with high as reference category; and low is analyzed with high as reference category.

Table 4 shows the adjusted multinomial logistic regression analysis results. Table S8, available online, provides additional regression analysis with varying reference classes. Differences are presented as odds ratios (OR) or adjusted odds ratios (aOR) along with 95% confidence intervals.

**Table 4:** Differences Between Classes Based on Adjusted Multinomial Logistic Regression Analysis

Independent variable	Confounders	Reference class	Class 1: Good mental health (n = 150)	Class 2: Severe mental distress (n = 35)	Class 3: Moderate mental strain (n = 32)	Class 4: Resilient (n = 41)
Age	Region of origin	1		1.10 (0.99-1.23)	1.07 (0.94-1.20)	1.09 (0.98-1.20)
		2			0.97 (0.83-1.12)	0.99 (0.86-1.12)
		3				1.02 (0.89-1.18)
		4				
Gender		1		1.07 (0.51-2.23)	1.72 (0.77-3.81)	1.27 (0.63-2.55)
Male		2			1.61 (0.60-4.31)	1.19 (0.48-4.31)
Ref: Female		3				0.74 (0.28-1.93)
		4				
Region of origin:		1		1.03 (0.44-2.41)	1.51 (0.65-3.53)	0.32 (0.11-0.96)
Sub-Saharan Africa		2			1.46 (0.50-4.31)	0.31 (0.09-1.11)
Ref: Middle East and North		3				0.21 (0.06-0.76)
Africa		4				
Asylum status:	Unaccompanied, region of origin, years in Sweden, parental education	1		5.59 (1.59-19.70)	2.24 (0.55-9.15)	0.70 (0.19-2.54)
Awaiting decision		2			0.40 (0.08-1.95)	0.13 (0.03-0.60)
Ref: Received asylum		3				0.31 (0.06-1.72)
		4				
Unaccompanied status	Age, gender, region of origin	1		10.80 (4.15-28.00)	2.75 (1.01-7.51)	3.00 (1.22-7.36)
Ref: Accompanied status		2			0.26 (0.08-0.83)	0.28 (0.09-0.83)
		3				1.09 (0.35-3.43)
		4				
Years in Sweden	Age, gender, unaccompanied	1		1.20 (0.99-1.46)	1.11 (0.92-1.34)	0.90 (0.75-1.08)
		2			0.92 (0.73-1.17)	0.75 (0.59-0.95)
		3				0.81 (0.64-1.03)
		4				
Parental education	Region of origin	1		1.68 (0.67-4.23)	2.51 (0.86-7.31)	1.50 (0.66-3.44)
Low		2			1.49 (0.41-5.47)	0.89 (0.30-2.71)
Ref: High		3				0.60 (0.18-2.06)
		4				
Parental education	Region of origin	1		1.09 (0.34-3.54)	2.58 (0.78-8.49)	1.34 (0.50-3.60)
Medium		2			2.36 (0.50-11.00)	1.23 (0.31-4.94)
Ref: High		3				0.52 (0.13-2.12)
		4				
Parental education	Region of origin	1		1.54 (0.51-4.69)	0.97 (0.35-2.72)	1.12 (0.42-2.99)
Low		2			0.63 (0.16-2.47)	0.73 (0.19-2.75)
Ref: Medium		3				1.15 (0.33-4.06)
		4				
ARQ Self	Unaccompanied, years in Sweden, exposure to violence, number of diagnoses	1		0.72 (0.44-1.17)	0.91 (0.62-1.33)	1.08 (0.77-1.52)
		2			1.27 (0.75-2.14)	1.50 (0.90-2.50)
		3				1.19 (0.76-1.84)
		4				
ARQ Family	Unaccompanied, exposure to violence, number of diagnoses	1		1.32 (0.78-2.22)	0.98 (0.66-1.45)	0.80 (0.58-1.11)
		2			0.74 (0.44-1.26)	0.61 (0.37-1.01)
		3				0.82 (0.54-1.24)
		4				
ARQ Peers	Years in Sweden, exposure to violence, number of diagnoses	1		0.66 (0.45-0.95)	1.03 (0.74-1.43)	0.85 (0.64-1.12)
		2			1.57 (1.04-2.36)	1.29 (0.90-1.87)
		3				0.83 (0.57-1.19)
		4				
ARQ School	Years in Sweden	1		1.04 (0.80-1.35)	1.04 (0.79-1.36)	0.86 (0.68-1.08)
		2			1.00 (0.71-1.40)	0.82 (0.60-1.13)
		3				0.83 (0.60-1.14)
		4				
ARQ Society	Unaccompanied, years in Sweden, exposure to violence, number of diagnoses	1		0.90 (0.59-1.37)	1.12 (0.77-1.62)	0.73 (0.54-1.00)
		2			1.25 (0.80-1.95)	0.82 (0.54-1.24)
		3				0.66 (0.44-0.97)
		4				
Exposure to number of different types of violence	Age, gender, unaccompanied, region of origin, years in Sweden	1		1.25 (1.15-1.36)	1.08 (1.01-1.16)	1.09 (1.02-1.17)
		2			0.87 (0.79-0.95)	0.88 (0.80-0.96)
		3				1.01 (0.93-1.10)
		4				
Exposure to sexual victimization	Age, gender, years in Sweden, unaccompanied	1		5.40 (2.16-13.50)	1.38 (0.51-3.78)	2.71 (1.19-6.19)
		2			0.26 (0.08-0.82)	0.50 (0.18-1.39)
		3				1.96 (0.65-5.94)
		4				
Exposure to child maltreatment	Age, region of origin, years in Sweden	1		8.12 (2.97-22.20)	1.66 (0.70-3.94)	1.47 (0.69-3.11)
		2			0.20 (0.06-0.68)	0.18 (0.06-0.58)
		3				0.89 (0.31-2.51)
		4				
Number of diagnoses	Unaccompanied, years in Sweden, exposure to violence	1		7.55 (3.82-14.90)	4.09 (2.12-7.89)	3.58 (1.88-6.84)
		2			0.54 (0.35-0.83)	0.48 (0.31-0.74)
		3				0.88 (0.55-1.40)
		4				
Sought treatment	Gender, parental education, years in Sweden, unaccompanied	1		7.34 (2.75-19.60)	3.23 (1.20-8.69)	1.50 (0.56-3.99)
		2			0.44 (0.14-1.44)	0.20 (0.07-0.64)
		3				0.46 (0.14-1.51)
		4				

Note: Data in Class 2, 3, and 4 columns are expressed as odds ratios or adjusted odds ratios with 95% CI in parentheses. ARQ = Adolescent Resilience Questionnaire.

The most notable results are presented below. Additional significant associations are described in Supplement 1, available online. No significant differences were found between the latent classes regarding gender. The unadjusted regression model indicated differences in parental education, but these differences were no longer apparent when the region of origin was factored into the adjusted model.

Participants in the Good Mental Health class experienced fewer types of violence (mean 8.52 compared to 12.31 to 17.71, aOR = 0.80-0.92, CI = 0.74-0.99) and were significantly more likely to have accompanied status (87.3% compared to 65.9% to 45.7%, aOR = 2.75-10.80, CI = 1.01-28.00) than those in other classes.

Individuals in the Severe Mental Distress class were significantly more likely to have unaccompanied status (54.3% compared to 12.7% to 34.1%, aOR = 3.60-10.80, CI = 1.30-18.00), more likely to have experienced more types of violence (mean 17.71 compared to 8.52 to 13.07, aOR = 1.14-1.25, CI = 1.04-1.36), and more likely to have faced child maltreatment (82.4% compared to 53.7% to 36.9%, aOR = 4.90-8.12, CI = 1.46-22.20), compared to all other classes. Moreover, when comparing to the Good Mental Health and Moderate Mental Strain classes, they showed a heightened prevalence of sexual victimization (55.9% compared to 16.1% and 21.9%, aOR = 5.40, CI = 2.16-13.50 and aOR = 3.90, CI = 1.21-12.60). Participants in this class were also more likely to still be waiting for a decision on their asylum application (37.1% compared to 10.0% and 14.6%, aOR = 5.59, CI = 1.59-19.70 and aOR = 7.98, CI = 1.66-38.40) in comparison to those in the Good Mental Health and Resilient classes.

The participants in the Moderate Mental Strain class were more likely to be unaccompanied than those in the Good Mental Health class (28.1% compared to 12.7%, aOR = 2.75, CI = 1.01-7.51), but less likely than participants in the Severe Mental Distress class (28.1% compared to 54.3%, aOR= 0.26, CI = 0.08-0.83). In addition, they showed a lower prevalence of exposure to various forms of violence, including more types of violence (mean 12.31 compared to 17.71, aOR = 0.87, CI = 0.79-0.95), sexual victimization (21.9% compared to 55.9%, aOR = 0.26, CI = 0.08-0.82), and child maltreatment (46.9% compared to 82.4%, aOR = 0.20, CI = 0.06-0.68) when compared to those in the Severe Mental Distress class.

Participants in the Resilient class were more likely to be unaccompanied than those in the Good Mental Health class (34.1% compared to 12.7%, aOR = 3.00, CI = 1.22-7.36), but less likely than participants in the Severe Mental Distress class (34.1% compared to 54.3%, aOR = 0.28, CI = 0.09-0.83). Moreover, those in the Resilient class were more likely to have experienced a wider range of violence and more sexual victimization compared to those in the Good Mental Health class (mean 13.07 compared to 8.52, aOR = 1.09, CI = 1.02-1.17, and 39.0% compared to 16.1%, aOR = 2.71, CI = 1.19-6.19). However, they reported a lower incidence of child maltreatment compared to participants in the Severe Mental Distress class (53.7% compared to 82.4%, aOR = 0.18, CI = 0.06-0.58).

## Discussion

To the best of our knowledge, this is the first study to use a quantitative person-centered method to investigate within-group heterogeneity in mental health among children and young adults with refugee backgrounds. The present study identified 4 distinct and clinically relevant classes. Social determinants of health, including being unaccompanied, asylum status, exposure to more types of violence, sexual victimization, and child maltreatment, distinguished the profiles.

The results further support the dual-factor model of mental health, demonstrating that individuals can experience a high symptom burden while showing good functioning and positive well-being. In addition, including well-being and general functioning as indicators helped to identify and distinguish low symptomatic groups. These groups have not been described in previously published person-centered analyses of mental health conducted in general populations of children.^24^

Most young people in our study belonged to the Good Mental Health class, despite the generally high levels of violence exposure in the sample and the fact that participants in this class had experienced an average of 8.52 different types of violence throughout their lives. This finding contradicts the current discourse that emphasizes the heavy burden of poor mental health among children and young adults with refugee backgrounds.^11^ Instead, it highlights their ability to cope, even under challenging circumstances, as shown in recent research.^47^ However, it is important to note that participants in this group still face challenges, such as exposure to violence, which are crucial to address in order to maintain the good mental health of this group. Furthermore, there is a risk of over-inclusion of participants in the Good Mental Health class in this study because of the potential stigma surrounding mental health, which might negatively affect their willingness to report mental health issues. This calls for caution in interpretation and emphasizes the necessity for replication.

The findings from the regression analysis indicated that accompanied status significantly predicts membership in the Good Mental Health class. This aligns with earlier studies showing that accompanied minors generally experience better mental health than unaccompanied individuals, despite some divergent results.^48^ This further highlights the potential protective role of parents after experiencing stressful events, and supports previous findings that have emphasized the importance of effective parenting and a positive parent–child relationship for favorable development following stressful experiences.^49^ In addition, having lived in Sweden for a shorter period emerged as another predictor of membership in this class. Given the mixed results found in a recent systematic review of Nordic studies,^50^ this finding should be interpreted cautiously.

The individuals who were the most significantly affected were those in the Severe Mental Distress class. Factors such as unaccompanied status, waiting for a decision on an asylum application, exposure to different forms of violence, sexual victimization, and child maltreatment predicted membership in this class. These findings highlight the significance of these combinations of risk factors, which have been studied individually in earlier research, often using variable-centered methods with symptom scores as outcomes.^51^, ^52^, ^53^ The results revealed that exposure to child maltreatment was significantly linked to a high likelihood of belonging to the Severe Mental Distress class compared to all other classes. This suggests that maltreatment during childhood may considerably affect the development of poor mental health in children and young adults with refugee backgrounds. This is consistent with previous research on the link between child maltreatment and poor mental health in high-income countries.^54^

The participants in the Moderate Strain class faced challenges in their daily lives but exhibited moderate well-being and no significant PTSS. High PTSS and good general functioning characterized the Resilient class despite substantial exposure to violence. In general, sexual victimization was associated with high PTSS scores, indicating membership in either the Severe Mental Distress or Resilient classes, thus confirming this type of violence as a risk factor for PTSS and PTSD diagnosis.^54^ Because of the limited sample size, further analysis of differences between the classes was not feasible. Additional differences likely exist, such as postmigration stressors,^55^ parental mental health issues, and parental exposure to adversity,^50^ which this study did not measure.

Treatment-seeking behavior and psychiatric diagnoses further validate the clinical relevance of the identified classes. Associations between these 2 variables and class memberships were in the anticipated directions. Participants in classes with better mental health had fewer psychiatric diagnoses and had sought treatment less often. A similar pattern was observed regarding the number of participants meeting the criteria for any psychiatric diagnosis (Table S6, available online). Unaccompanied status was identified as a risk factor for membership in the Severe Mental Distress class, aligning with findings from previous Swedish studies that indicated that children with refugee backgrounds arriving unaccompanied in Sweden have a higher prevalence of service use compared to most of their peers.^56^ However, despite the severity of poor mental health in this class, only 62.5% of participants sought treatment for any mental health issue. The current study cannot explain this relatively low rate of treatment-seeking behavior. Previous Swedish research has shown that children and young adults with refugee backgrounds face significant barriers when trying to access psychiatric care.^56^ Interestingly, we found no differences when analyzing age as a continuous and categorical variable or when including age group (child/young adult) as a covariate, highlighting the need for more research on young people transitioning between child and adult services with shifting legal rights and responsibilities, such as those related to asylum processes.^47^

Health care systems with limited resources need to differentiate between various mental health needs, even when the overall picture suggests high risks and rates of psychiatric symptoms for specific populations, such as individuals with refugee backgrounds.^12^^,^^13^ The findings of this study indicated that some individuals indeed exhibited severe symptoms, functional impairments, and low well-being, necessitating specialized psychiatric care; however, most did not require interventions. Compounding the issue, individuals in the Moderate Mental Strain category faced coping challenges but did likely not need specialized care. This group could benefit from community- and school-based interventions to enhance functioning. Finally, the Resilient group showed the ability to function well in daily life despite considerable PTSD symptoms. These individuals may benefit from evidence-based trauma interventions, such as trauma-focused cognitive behavioral therapy, but delivered in a concise and focused manner.

In general, person-centered methods involve large sample sizes, with a sample size of 300 participants or fewer typically regarded as relatively small.^44^ This necessitates caution in interpretation and conclusions. Furthermore, the sample size influences the statistical power to detect differences between classes in regression analysis, and the identified associations risk being overestimated. In addition, it was not feasible to assess the reliability of classes through split samples or to conduct comparability studies using similar indicators. As a result, replicating the findings in future studies is essential. Moreover, the sample was not representative, limiting the generalizability of the findings.

Most questionnaires used in this study have not been extensively validated or used in populations with refugee backgrounds, which limits their validity. The use of interpreters in the interviews may have introduced reporting biases, but it can also be viewed as a strength, as it facilitated clarifications during the interviews. Furthermore, the study relied solely on PTSS as a symptom indicator for the classes, neglecting other significant clusters, such as depressive and psychotic symptoms. This approach may have constrained our understanding of the heterogeneity within the sample. Finally, increased stigma surrounding mental health has been previously noted in populations with refugee backgrounds, which may have resulted in the under-reporting of symptoms in our sample.

The study also has several strengths. To our knowledge, this is one of the few studies on children and young adults with refugee backgrounds that have used state-of-the-art interviews, encompassed a broad range of nationalities, and examined both accompanied and unaccompanied individuals recruited nationally while focusing on a wide range of mental health issues. The methodology used is a novel approach that enables person-centered analysis of within-group variation and integrates multiple indicators, including positive measures, to evaluate mental health and its dimensions, as well as to identify combinations of health determinants.

Children and young adults with refugee backgrounds can be categorized into distinct classes based on a set of clinically relevant indicators of mental health. Focusing solely on those individuals at the highest risk for poor mental health may overlook many who are mentally healthy and those who need more targeted support. Future research should aim to replicate findings and to examine additional predictive variables at the family and societal levels. In general, person-centered analytic approaches can enhance the understanding of the varied mental health needs within diverse groups, thus providing further guidance on assessments as well as tailored support and interventions.

## CRediT authorship contribution statement

**Johan Andersson:** Writing – original draft, Investigation, Formal analysis, Data curation, Conceptualization. **Hongru Zhai:** Writing – review & editing, Investigation, Formal analysis, Data curation. **Reeta Kankaanpää:** Writing – review & editing, Investigation, Formal analysis. **Carolina Bråhn:** Writing – review & editing, Investigation, Data curation. **Erica Mattelin:** Writing – review & editing, Data curation. **Kirsi Peltonen:** Writing – review & editing, Investigation. **Ann-Charlotte Münger:** Writing – review & editing, Investigation. **Laura Korhonen:** Writing – review & editing, Supervision, Investigation, Funding acquisition, Conceptualization.

## Supplemental Material

Supplemental Material l
