# Supplementary material for: Latent Profile Analysis of Mental Health Among Children and Young Adults With Refugee Backgrounds
Source: JAACAP Open. 2025 Jun 16;3(4):1188–201. doi: 10.1016/j.jaacop.2025.06.003 (PMC12684649; doi:10.1016/j.jaacop.2025.06.003)
Supplement: Supplemental Material — l [file mmc1.docx]

**Supplementary materials**

| **Table of Contents** | **Page** |
| --- | --- |
| Supplement 1 | 1 |
| Table S1: Correlations between indicators in total sample | 6 |
| Table S2: Correlations between indicators in each latent class | 6 |
| Table S3: Fit indices for model 1 | 7 |
| Table S4: Fit indices for model 2 | 8 |
| Figure S1: Profile plot for model 2 with 3 classes | 8 |
| Table S5: Fit indices for model 6 | 9 |
| Figure S2: Profile plot for model 6 with 3 classes | 9 |
| Table S6: Descriptives with additional variables for total sample and classes and differences between classes based on unadjusted regression model | 10 |
| Table S7: Differences between classes based on adjusted multinominal logistic regression analysis with additional variables | 15 |
| Table S8: Additional regression analysis with varying reference classes | 18 |
| References | 19 |

**Supplement 1**

**Methods**

***Procedure***

Regarding consent, if the participants were over 15, they could provide consent independently. If they were younger, their legal guardians provided the consent.

***Transformation of covariates for regression analysis***

The country of origin was transformed into a region of origin, which included the following categories: North Africa and the Middle East, Sub-Saharan Africa, and Other. Asylum status was dichotomized into two categories (awaiting decision/decision received) for regression analysis. Parental education was coded into low, medium, and high categories based on the Swedish standard education classification^1^, and the mother’s and father’s education was combined into a single variable by using the highest education level of either parent as the final classification. Many study participants expressed uncertainty about their race and ethnicity, which is why data is not reported.

***Measures***

The Juvenile Victimization Questionnaire (JVQ) has been extensively validated and used in studies measuring exposure to violence among children and adolescents in a range of countries, including the United Kingdom, the United States, China, Switzerland and Pakistan.^2-4^ It has also been used in studies with samples which has included young adults.^5,6^ The JVQ has not yet been validated or extensively utilized in populations with refugee backgrounds. The following questions were added to the questionnaire for this study.

- Have you experienced a serious natural disaster such as a flood, tornado, hurricane, earthquake or fire?
- Serious accident or injury such as, car/bike accident, dog bite or sports injury?
- Stressful or frightening medical examination?
- Sudden or violent death of someone close to you?
- Growing up, have you experienced poverty in your family?
- Have you had to work to support yourself or your family?
- Have you had to be separated from your parents or someone important to you because of war or other disturbances?
- Have you been captured or imprisoned in any way?
- Have you been involved in human trafficking for sexual purposes?
- Have you been trafficked for the removal of organs?
- Have you been trafficked for military service?
- Have you been trafficked for forced labour (i.e. you have been forced to work without pay or for very little pay. This also includes domestic work, if it was more extensive than is usual for someone of your age)?
- Have you been trafficked for any other purpose, in a situation of distress for the victim, e.g. begging or committing a crime? Or something else similar that we have not asked about? To interviewer: if so, please write this in the comment section.

From the JVQ, this study used scores on exposure to different types of violence and module scores on child maltreatment and sexual victimization.

The Child and Adolescent Trauma Screen version 1 (CATS-1) has previously been used in German study with a population of children and adolescents with refugee backgrounds (α=0.81).^7^ Additionally, its validity and reliability has been tested across several languages and accompanying populations.^8^

The Posttraumatic Stress Disorder Checklist for DSM-5 (PCL-5) has previously been used in a study on refugees in Germany (α=0.93-0.97)^9^, and more recently among displaced Palestinian young adults from Gaza (α=0.92)^10^, and Syrian refugee women in Jordan (α=0.94).^11^

The WHO-5 has previously been used in a study investigating mental illness in Syrian refugees resettled in Sweden (α=0.94).^12^ The questionnaire was also used in a study on refugees resettled in Denmark^13^, and recently in a study on adolescent girls in Palestinian refugee camps (α=0.81).^14^

The Adolescent Resilience Questionnaire (ARQ) has been translated into and tested in multiple different languages, including Swedish^15^. However, it has yet to be validated in a sample of children and adolescents with refugee backgrounds and has mainly been used with adolescents under the age of 19. For this study, we selected the following items from the ARQ. The highest loading item for each subscale (Self, Family, Peers, School and Society) based on previous studies^15,16^ was selected for use in the interview.

ARQ item Self:
*I feel confident that I can handle whatever comes my way.*

ARQ item Family:
*I enjoy spending time with my family.*

ARQ item Peers:
*I have a friend I can trust with my private thoughts and feelings.*

ARQ item School
*My teachers are caring and supportive of me.*

ARQ item Society
*I trust people in my neighborhood.*

The Mini International Neuropsychiatric Interview for Children and Adolescents 6.0 (MINI-KID) has been validated in Sweden^17^ and previously been used and rigorously tested in a population of Syrians with refugee backgrounds residing in Lebanon.^18^ The Mini International Neuropsychiatric Interview 7.0 (MINI) has previously been used in several different refugee populations.^19,20^

***Reliability***

Internal consistency:

WHO-5

α = 0.86

CATS-1

Total scale: α = 0.94

Cluster B: α = 0.87

Cluster C: α = 0.72

Cluster D: α = 0.84

Cluster E: α = 0.80

PCL-5

Total scale: α = 0.90

Cluster B: α = 0.75

Cluster C: α = 0.67

Cluster D: α = 0.77

Cluster E: α = 0.80

***Correlation analysis***

Correlation analysis was performed to determine the overlap between the indicators in the total sample and in the identified classes. Low to moderate correlations were identified in the total sample. One significant low correlation was identified in class 2 (Table S1 and Table S2).

***Models fit and selection of final solution***

Concerning models, six different types were fit; model 1 where variances were estimated to be equal across profiles and covariances constrained to be zero, model 2 where variances could vary and covariances were fixed to 0, model 3 where variances and covariances were estimated to be equal, model 4 with varying variances and equal covariances, model 5 with equal variances and varying covariances and model 6 where the variances and the covariances were allowed be freely estimated across profiles. All models were run multiple times and fit indices evaluated along with interpretability of plots to select promising solutions. The stability of the chosen promising solutions was then assessed via simulation, where analysis was run with 100 iterations. The most stable solutions were further assessed based on a combination of fit indices, plots and theoretical interpretability and meaningfulness.

***Multinomial regression analysis equations***

Unadjusted model:

$$\log\left( \frac{P\left( Class=k \right)}{P\left( Class=ref \right)} \right)= \beta_{0}^{(k)}+\beta_{1}^{(k)}X$$

Where:

- k represents each non-reference class.
- X is the independent variable.
- β are the regression coefficients for class k.

Adjusted model:

$$\log\left( \frac{P\left( Class=k \right)}{P\left( Class=ref \right)} \right)= \beta_{0}^{(k)}+\beta_{1}^{(k)}X+\beta_{2}^{(k)}C_{1}+\beta_{3}^{(k)}C_{2}+\cdots+\beta_{m}^{(k)}C_{M}$$

Where:

- *C* are the confounders defined for each independent variable

**Results**

***Differences between classes***

Participants in the Good Mental Health Class met criteria for fewer types of psychiatric diagnoses (mean 0.11 compared to 0.63 to 2.39, aOR ranging from 0.13 to 0.28, CI ranging from 0.07 to 0.53) compared to all other classes.

Participants in the Severe Mental Distress class were more likely to meet criteria for a higher number of psychiatric diagnoses (mean 2.39 compared to 0.11 to 0.77, aOR ranging from 1.85 to 7.57, CI ranging from 1.20 to 15.00) compared to all other classes. They had sought treatment for psychological difficulties to a greater extent (57.1% compared to 12.0% and 22.0%, aOR 7.34, CI 2.75-19.60 and aOR 4.90, CI 1.56-15.30) compared to participants in the Good Mental Health and Resilient classes. They to a lower extent felt that they had a close friend with whom they could share important things (ARQ item Peers) (mean 3.15 compared to 4.23, aOR 0.65, CI 0.45-0.95) and had lived in Sweden for a longer period (mean 3.55 compared to 2.44, aOR 1.20, CI 0.99-1.46) compared to participants in the Good Mental Health class.

Participants belonging to the Moderate Mental Strain class felt to a higher extent that they had close friends to share important things with than participants in the Severe Mental Distress class (mean 4.13 compared to 3.15, aOR 1.57, CI 1.04-2.35).

Participants in the Resilient class had lived in Sweden for a shorter period compared to participants in the Severe Mental Distress class (mean 2.74 compared to 3.55, aOR 0.75, CI 0.59-0.95). They were less likely to come from Sub-Saharan Africa as compared to those in the Good Mental Health class (9.8% compared to 25.3%, OR 0.32, CI 0.11-0.96) and the Moderate Mental Strain class (9.8% compared to 31.3%, OR 4.74, CI 0.06-0.76). Additionally, they rated their neighborhoods as less safe (ARQ item Society) in comparison to those in the Good Mental Health class (mean 2.88 compared to 3.64, aOR 0.73, CI 0.54-1.00) and the Moderate Mental Strain class (mean 2.88 compared to 3.43, aOR 0.66, CI 0.44-0.97). Furthermore, they exhibited a higher prevalence of psychiatric diagnoses in comparison to those in the Good Mental Health class (aOR 3.59, CI 1.88-6.86).

**Table S1: Correlations between indicators in total sample**

| **Measure** | 1 | 2 | 3 |
| --- | --- | --- | --- |
| 1. GAF/C-GAS | - |  |  |
| 2. WHO-5 | 0.50^*^ | - |  |
| 3. CATS-1/PCL-5 | 0.56^*^ | 0.38^*^ | - |

Note: Correlations presented as Spearman’s rank-order correlations

**^*^,** p < 0.05

**Table S2: Correlations between indicators in each latent class**

| **Correlation variables** | **Class** | | | |
| --- | --- | --- | --- | --- |
|  | **1** | **2** | **3** | **3** |
| GAF/C-GAS – WHO-5 | 0.08 | 0.27^*^ | -0.18 | 0.14 |
| WHO-5 – CATS-1/PCL-5 | -0.01 | 0.02 | 0.11 | 0.00 |
| CATS-1/PCL-5 – GAF/C-GAS | 0.07 | -0.13 | 0.22 | 0.00 |

Note: Correlations presented as Spearman’s rank-order correlations

**^*^,** p < 0.05

**Table S3: Fit indices for model 1**

| **Model** | **Classes** | **AIC** | **BIC** | **Entropy** | **Prob**  **min** | **Prob**  **max** | **N**  **min** | **N**  **max** | **BLRT**  **p** |
| --- | --- | --- | --- | --- | --- | --- | --- | --- | --- |
| 1 | 1 | 2217.33 | 2238.64 | 1.00 | 1.00 | 1.00 | 1.00 | 1.00 |  |
| 1 | 2 | 1966.76 | 2002.28 | 0.89 | 0.93 | 0.98 | 0.24 | 0.76 | 0.01 |
| 1 | 3 | 1920.52 | 1970.26 | 0.86 | 0.80 | 0.98 | 0.13 | 0.67 | 0.01 |
| **1** | **4** | **1877**.**34** | **1941**.**30** | **0.88** | **0.83** | **0.97** | **0.12** | **0.58** | **0.01** |
| 1 | 5 | 1874.93 | 1953.10 | 0.87 | 0.77 | 0.97 | 0.03 | 0.57 | 0.05 |
| 1 | 6 | 1874.12 | 1966.49 | 0.85 | 0.56 | 0.97 | 0.04 | 0.57 | 0.13 |

Note: AIC = Akaike’s information criterion; BIC = Bayesian information criterion; BLRT = bootstrapped likelihood ratio test; Prob = Probability.

**Table S4: Fit indices for model 2**

| **Model** | **Classes** | **AIC** | **BIC** | **Entropy** | **Prob**  **min** | **Prob**  **max** | **N**  **min** | **N**  **max** | **BLRT**  **p** |
| --- | --- | --- | --- | --- | --- | --- | --- | --- | --- |
| 2 | 1 | 2214.55 | 2235.87 | 1.00 | 1.00 | 1.00 | 1.00 | 1.00 |  |
| 2 | 2 | 1875.34 | 1921.53 | 0.83 | 0.95 | 0.97 | 0.47 | 0.53 | 0.01 |
| 2 | 3 | 1771.73 | 1842.78 | 0.86 | 0.93 | 0.98 | 0.11 | 0.46 | 0.01 |
| 2 | 4 | 1755.20 | 1851.13 | 0.84 | 0.85 | 0.98 | 0.10 | 0.43 | 0.01 |
| 2 | 5 | 1726.75 | 1847.55 | 0.83 | 0.87 | 0.99 | 0.10 | 0.36 | 0.01 |
| 2 | 6 | 1735.68 | 1881.35 | 0.89 | 0.90 | 0.98 | 0.03 | 0.37 | 0.12 |

Note: AIC = Akaike’s information criterion; BIC = Bayesian information criterion; BLRT = bootstrapped likelihood ratio test; Prob = Probability.

**Figure S1: Profile plot for model 2 with 3 classes**

**
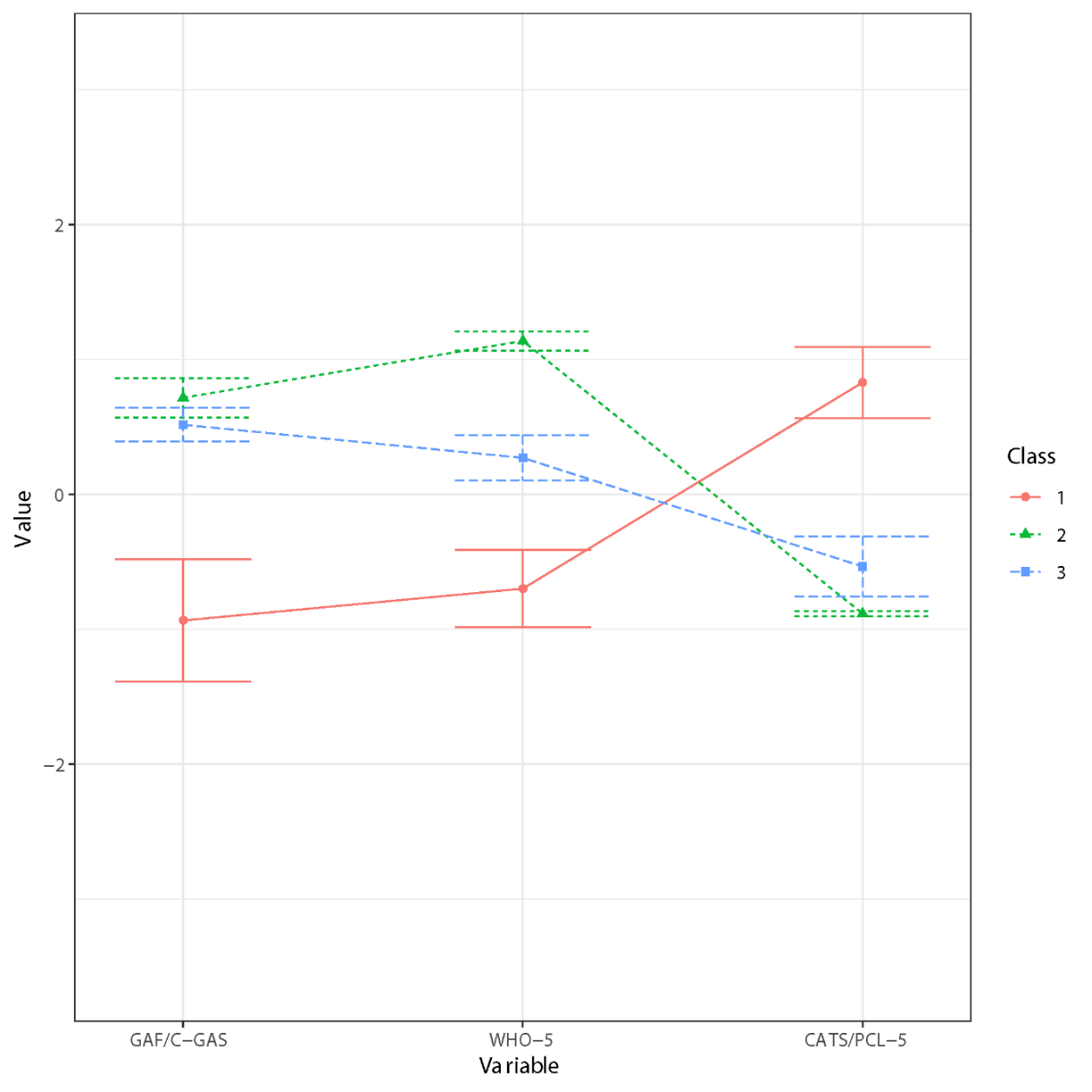
**

Note: Values are standardized for ease of comparison. CATS-1 = Child and Adolescent Trauma Screen version 1; C-GAS = Children’s Global Assessment Scale; GAF = Global Assessment of Functioning; PCL-5 = Posttraumatic Stress Disorder Checklist for DSM-5; WHO-5 = World Health Organization Well-being Index.

**Table S5: Fit indices for model 6**

| **Model** | **Classes** | **AIC** | **BIC** | **Entropy** | **Prob**  **min** | **Prob**  **max** | **N**  **min** | **N**  **max** | **BLRT**  **p** |
| --- | --- | --- | --- | --- | --- | --- | --- | --- | --- |
| 6 | 1 | 2002.96 | 2034.94 | 1.00 | 1.00 | 1.00 | 1.00 | 1.00 |  |
| 6 | 2 | 1852.07 | 1919.57 | 0.72 | 0.90 | 0.95 | 0.50 | 0.50 | 0.01 |
| 6 | 3 | 1775.51 | 1878.54 | 0.80 | 0.90 | 0.98 | 0.14 | 0.45 | 0.01 |
| 6 | 4 | 1771.41 | 1909.97 | 0.75 | 0.80 | 0.99 | 0.14 | 0.38 | 0.17 |
| 6 | 5 | 1773.60 | 1947.70 | 0.81 | 0.85 | 0.99 | 0.11 | 0.31 | 0.40 |

Note: AIC = Akaike’s information criterion; BIC = Bayesian information criterion; BLRT = bootstrapped likelihood ratio test; Prob = Probability.

**Figure S2: Profile plot for model 6 with 3 classes**

**
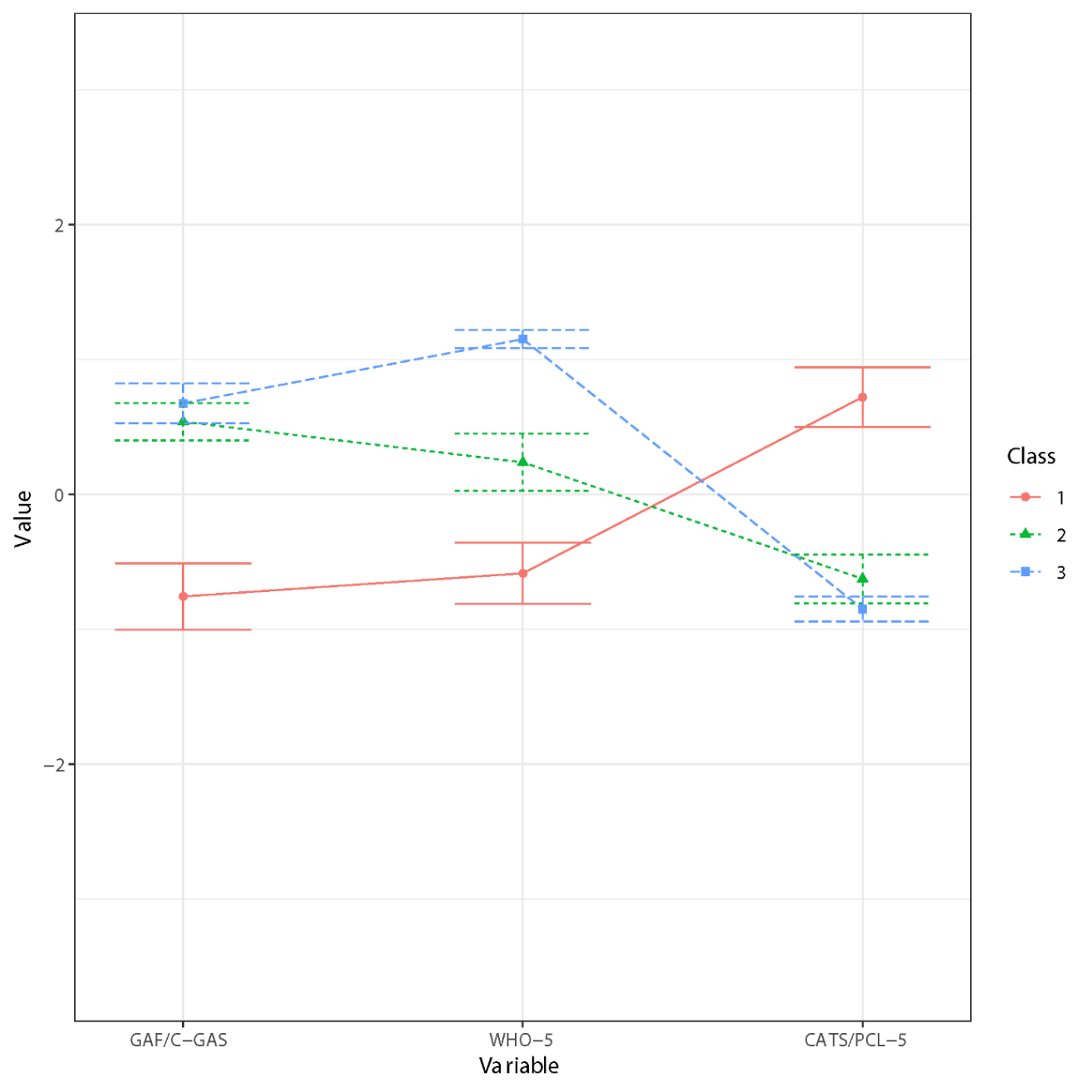
**

Note: Values are standardized for ease of comparison. CATS-1 = Child and Adolescent Trauma Screen version 1; C-GAS = Children’s Global Assessment Scale; GAF = Global Assessment of Functioning; PCL-5 = Posttraumatic Stress Disorder Checklist for DSM-5; WHO-5 = World Health Organization Well-being Index.

**Table S6: Descriptives with additional variables for total sample and classes and differences between classes based on unadjusted regression model.**

| **Independent variable** | **Total**  **(n = 258)** | **Class 1 Good mental health**  **(n = 150)** | **Class 2 Severe mental distress**  **(n = 35)** | **Class 3 Moderate mental strain**  **(n = 32)** | **Class 4 Resilient**  **(n = 41)** | **Significant differences between classes (p < 0.05)** |
| --- | --- | --- | --- | --- | --- | --- |
| Age, years, mean (SD)  Min/max  Missing | 18.21 (3.75)  12/25  0 | 17.18 (3.79)  12/25  0 | 18.83 (3.30)  14/25  0 | 18.28 (3.66)  12/25  0 | 19.22 (3.86)  12/25  0 | C1 < C4 |
| Age group, n (%)  Children  Young adults  Missing | 131 (50.8%)  127 (49.2%)  0 | 82 (54.7%)  68 (45.3%)  0 | 16 (45.7%)  19 (54.3%)  0 | 18 (56.2%)  14 (43.8%)  0 | 15 (36.6%)  26 (63.4%)  0 | C1 < C4 |
| Age categories, n (%)^a^  12-14  15-17  18-21  22-25  Missing | 46 (17.8%)  77 (29.8)  70 (27.1%)  65 (25.2%)  0 | 34 (22.7%)  41 (27.3%)  41 (27.3%)  34 (22.7%)  0 | 3 (8.6%)  13 (37.1%)  10 (28.6%)  9 (25.7%)  0 | 4 (12.5%)  14 (43.8%)  6 (18.8%)  8 (25%)  0 | 5 (12.2%)  9 (22.0%)  13 (31.7%)  14 (34.1%)  0 |  |
| Gender, female, n (%)  Missing | 115 (44.6%)  0 | 71 (47.3%)  0 | 16 (45.7%)  0 | 11 (34.40%)  0 | 17 (41.50%)  0 |  |
| Area of origin, n (%):  Middle East & Africa  Sub-Saharan Africa  Other*  Missing | 189 (73.5%)  61 (23.7%)  7 (2.7%)  1 | 109 (72.7%)  38 (25.3%)  3 (2%)  0 | 25 (71.4%)  9 (25.7%)  1 (2.9%)  0 | 19 (61.3%)  10 (32.2%)  2 (6.5%)  1 | 36 (87.8%)  4 (9.8%)  1 (2.4%)  0 | C4 < C1, C3 |
| Asylum status:^c^  Decision received  Awaiting decision  Appealing  Do not know*  Missing | 200 (77.5%)  36 (14.0%)  4 (1.6%)  18 (7.0%)  0 | 120 (80.0%)  15 (10%)  0 (0%)  15 (10%)  0 | 22 (62.9%)  11 (31.4%)  2 (5.7%)  0 (0%)  0 | 24 (75.0%)  5 (15.6%)  1 (3.1%)  2 (6.3%)  0 | 34 (82.9%)  5 (12.2%)  1 (2.4%)  1 (2.4%)  0 | C2 > C1, C4 |
| Time in Sweden, months, mean (SD)  Min/max  Missing, n | 32.58 (35.35)  1/161  2 | 29.26 (34.40)  1/161  2 | 42.57 (38.00)  1/111  0 | 36.66 (42.90)  1/144  0 | 32.85 (28.60)  1/92  0 | C2 > C1 |
| Time in Sweden, years, Mean (SD)  Min/max Missing, n | 2.71 (2.95)  0.08/13.42  2 | 2.44 (2.87)  0.08/13.42  2 | 3.55 (3.16)  0.08/9.25  0 | 3.05 (3.57)  0.08/12.00  0 | 2.74 (2.39)  0.08/7.67  0 | C2 < C1 |
| Unaccompanied status, n (%)  Missing | 61 (23.6%)  0 | 19 (12.7%)  0 | 19 (54.3%)  0 | 9 (28.1%)  0 | 14 (34.1%)  0 | C1 < C2, C3, C4  C2 > C3, C4 |
| Parental education, (%)^d^  High  Medium  Low  Missing | 94 (38.1%)  49 (19.8%)  104 (42.1%)  11 | 59 (40.7%)  28 (19.3%)  58 (40.0%)  5 | 11 (34.3%)  5 (15.6%)  16 (50.0%)  3 | 9 (30.0%)  7 (23.3%)  14 (46.7%)  2 | 15 (37.5%)  9 (22.5%)  16 (40.0%)  1 | C1 > C2  C1 < C2  C1 < C2 |
| ARQ items, mean (SD)  ARQ Self  Min/max  Missing  ARQ Family  Min/max  Missing  ARQ Peers  Min/max  Missing  ARQ School  Min/max  Missing  ARQ Society  Min/max  Missing | 3.67 (1.20)  1/5  9  4.10 (1.16)  1/5  6  4.00 (1.40)  1/5  3  3.03 (1.40)  1/5  7  3.40 (1.36)  1/5  9 | 3.69 (1.27)  1/5  5  4.21 (1.09)  1/5  5  4.23 (1.25)  1/5  1  3.08 (1.49)  1/5  5  3.64 (1.23)  1/5  7 | 3.43 (1.16)  1/5  0  3.89 (1.35)  1/5  0  3.15 (1.58)  1/5  1  3.12 (1.49)  1/5  1  3.02 (1.56)  1/5  0 | 3.67 (0.96)  1/5  2  4.07 (1.09)  1/5  1  4.13 (1.31)  1/5  1  3.13 (1.34)  1/5  1  3.43 (1.36)  1/5  2 | 3.87 (1.10)  1/5  2  3.88 (1.29)  1/5  0  3.81 (1.58)  1/5  0  2.73 (1.60)  1/5  0  2.88 (1.49)  1/5  0 | C1 > C2; C2 < C3  C1 > C2, C4 |
| Exposure to number of different types of violence, mean (SD)  Min/max  Missing | 10.97 (7.22)  0/34  1 | 8.52 (6.32)  0/25  1 | 17.71 (7.41)  4/34  0 | 12.31 (7.39)  0/25  0 | 13.07 (5.33)  5/28  0 | C1 < C2, C3, C4  C2 > C3, C4 |
| Exposure to sexual violence, yes, n (%)  Missing | 66 (25.8%)  2 | 24 (16.1%)  1 | 19 (55.9%)  1 | 7 (21.9%)  0 | 16 (39.0%)  0 | C1 < C2, C4  C2 > C3 |
| Exposure to child maltreatment, yes, n (%)  Missing | 120 (46.9%)  2 | 55 (36.9%)  1 | 28 (82.4%)  1 | 15 (46.9%)  0 | 22 (53.7%)  0 | C1 < C2  C2 > C3, C4 |
| Depression, n (%)  Missing | 15 (6.2%)  15 | 1 (0.7%)  5 | 8 (26.7%)  6 | 2 (6.9%)  3 | 4 (10.0%)  1 | Sample too small |
| Dystemi^e^, n (%)  Missing | 5 (4.3%)  12 | 1 (1.3%)  2 | 3 (27.3%)  5 | 0 (0%)  4 | 1 (7.1%)  1 | Sample too small |
| Suicidal thoughts, n (%)  Missing | 33 (14.2%)  26 | 3 (2.2%)  13 | 19 (59.4%)  3 | 5 (20.8%)  8 | 6 (15.4%)  2 | Sample too small |
| Suicide attempts, n (%)  Missing | 7 (3.2%)  38 | 0 (0%)  15 | 4 (14.8%)  8 | 0 (0%)  11 | 3 (8.1%)  4 | Sample too small |
| NSSI, n (%)  Missing | 17 (7.6%)  33 | 3 (2.3%)  17 | 9 (31.0%)  6 | 2 (8.3%)  8 | 3 (7.7%)  2 | Sample too small |
| GAD, n (%)  Missing | 10 (4.2%)  18 | 1 (0.7%)  6 | 5 (17.2%)  6 | 1 (3.9%)  6 | 3 (7.3%)  0 | Sample too small |
| OCD, n (%)  Missing | 9 (3.8%)  18 | 0 (0%)  8 | 8 (27.6%)  6 | 1 (3.6%)  4 | 0 (0%)  0 | Sample too small |
| Social anxiety, n (%)  Missing | 3 (1.2%)  13 | 0 (0%)  5 | 2 (6.9%)  6 | 0 (0%)  3 | 1 (2.4%)  0 | Sample too small |
| Specific phobia, n (%)  Missing | 4 (3.4%)  14 | 2 (2.6%)  5 | 0 (0%)  6 | 1 (6.7%)  3 | 1 (6.7%)  0 | Sample too small |
| Panic disorder, n (%)  Missing | 11 (4.4%)  10 | 1 (0.7%)  3 | 8 (25.9%)  4 | 1 (3.5%)  3 | 1 (2.4%)  0 | Sample too small |
| Any anxiety disorder, n (%)  Missing | 29 (11.7%)  9 | 4 (2.7%)  2 | 15 (48.4%)  4 | 4 (13.8%)  3 | 6 (14.6%)  0 | Sample too small |
| ODD^f^, n (%)  Missing | 3 (2.5%)  13 | 0 (0%)  3 | 1 (10.0%)  6 | 2 (13.3%)  3 | 0 (0%)  1 | Sample too small |
| ADHD^f^, n (%)  Missing | 1 (0.8%)  12 | 0 (0%)  3 | 0 (0%)  5 | 1 (6.7%)  3 | 0 (0%)  1 | Sample too small |
| Conduct disorder^f^, n (%)  Missing | 1 (0.8%)  13 | 0 (0%)  3 | 0 (0%)  6 | 1 (6.7%)  3 | 0 (0%)  1 | Sample too small |
| Eating disorder  Missing | 3 (1.2%)  17 | 0 (0%)  5 | 1 (3.6%)  7 | 1 (3.7%)  5 | 1 (2.4%)  0 | Sample too small |
| Psychotic symptoms  Missing | 13 (5.6%)  24 | 1 (0.7%)  9 | 7 (24.1%)  6 | 4 (16.0%)  7 | 1 (2.6%)  2 | Sample too small |
| Alcohol disorder  Missing | 5 (2.2%)  30 | 2 (1.4%)  11 | 3 (12.0%)  10 | 0 (0%)  7 | 0 (0%)  2 | Sample too small |
| Substance disorder  Missing | 5 (2.1%)  14 | 1 (0.7%)  4 | 1 (3.5%)  6 | 2 (6.7%)  2 | 1 (2.6%)  2 | Sample too small |
| Any diagnosis, n (%)  Missing | 71 (28.1%)  5 | 13 (8.8%)  2 | 25 (75.8%)  2 | 15 (48.4%)  1 | 18 (43.9%)  0 | C1 < C2, C3, C4  C2 > C3, C4 |
| Externalizing diagnosis^f^, n (%)  Missing | 4 (3.4%)  12 | 0  3 | 1 (9.1%)  5 | 3 (20.3%)  3 | 0  1 | Sample too small |
| Internalizing diagnosis, n (%)  Missing | 46 (18.3%)  7 | 6 (4.1%)  2 | 21 (65.6%)  3 | 8 (26.7%)  2 | 11 (26.8%)  0 | C1 < C2, C3, C4  C2 > C3, C4 |
| Number of diagnoses, mean (SD)  Min/max  Missing | 0.57 (1.18)  0/7  5 | 0.11 (0.39)  0/3  2 | 2.39 (1.95)  0/7  2 | 0.77 (1.09)  0/4  1 | 0.63 (0.86)  0/3  0 | C1 < C2, C3, C4  C2 > C3, C4 |
| Multimorbidity, n (%)  Missing | 33 (13.0%)  5 | 2 (1.4%)  2 | 20 (60.6%)  2 | 5 (16.1%)  1 | 6 (14.6%)  0 | Sample too small |
| Sought treatment, yes, n (%)  Missing | 57 (22.7%)  7 | 18 (12.1%)  1 | 20 (62.5%)  3 | 10 (34.5%)  3 | 9 (22%)  0 | C1 < C2, C3  C2 > C3, C4 |

a. Analyzed with 12-14 as the reference category.
b. Not included in analysis of differences between classes
c. Asylum status was dichotomized into two categories (awaiting decision/decision received) for analysis of differences between classes.
d. High is analyzed with low or medium as reference category, Medium is analyzed with high as reference category, and Low is analyzed with high as reference category
e. Percentages calculated from young adult group
f. Percentages calculated from children group

Note: Missing values are reported for individual items, except for exposure to number of different types of violence, exposure to sexual victimization and exposure to child maltreatment for which missing values indicates the whole scale. ADHD = attention deficit hyperactivity disorder; ARQ = Adolescent Resilience Questionnaire; GAD = generalized anxiety disorder; NSSI = non-suicidal self-injury; OCD = obsessive–compulsive disorder; ODD = oppositional defiant disorder; SD = standard deviation.

**Table S7: Differences between classes based on adjusted multinominal logistic regression analysis with additional variables.**

| **Independent variable** | **Covariates/Confounders** | **Reference class** | **Class 1 Good mental health**  **(n = 150)** | **Class 2 Severe mental distress**  **(n = 35)** | **Class 3 Moderate mental strain**  **(n = 32)** | **Class 4 Resilient**  **(n = 41)** |
| --- | --- | --- | --- | --- | --- | --- |
| Age | Region | 1  2  3  4 | - | 1.10 (0.99-1.23)  - | 1.07 (0.94-1.20)  0.97 (0.83-1.12)  - | 1.09 (0.98-1.20)  0.99 (0.86-1.12)  1.02 (0.89-1.18)  - |
| Age groups  Young adults  Ref: Children | Region | 1  2  3  4 | - | 1.77 (0.71-4.44)  - | 1.23 (0.46-3.26)  0.70 (0.20-2.37)  - | 1.61 (0.72-3.60)  0.91 (0.30-2.74)  1.31 (0.41-4.12)  - |
| Gender  Male  Ref: Female |  | 1  2  3  4 | - | 1.07 (0.51-2.23)  - | 1.72 (0.77-3.81)  1.61 (0.60-4.31)  **-** | 1.27 (0.63-2.55)  1.19 (0.48-4.31)  0.74 (0.28-1.93)  - |
| Area of origin:  Sub-Saharan Africa  Ref: Africa & ME |  | 1  2  3  4 | - | 1.03 (0.44-2.41)  - | 1.51 (0.65-3.53)  1.46 (0.50-4.31)  - | 0.32 (0.11-0.96)  0.31 (0.09-1.11)  0.21 (0.06-0.76)  - |
| Asylum status:  Awaiting decision  Ref: Received asylum | Unaccompanied, Region, Time in Sweden, Parental education | 1  2  3  4 | - | 5.59 (1.59-19.70)  - | 2.24 (0.55-9.15)  0.40 (0.08-1.95)  - | 0.70 (0.19-2.54)  0.13 (0.03-0.60)  0.31 (0.06-1.72)  - |
| Unaccompanied  Ref: Accompanied | Age, Gender, Region | 1  2  3  4 | - | 10.80 (4.15-28.00)  - | 2.75 (1.01-7.51)  0.26 (0.08-0.83)  - | 3.00 (1.22-7.36)  0.28 (0.09-0.83)  1.09 (0.35-3.43)  - |
| Time in Sweden, months | Age, Gender, Unaccompanied | 1  2  3  4 | - | 1.02 (1.00-1.03)  - | 1.01 (0.99-1.02)  0.99 (0.97-1.01)  - | 0.99 (0.98-1.01)  0.98 (0.96-1.00)  0.98 (0.96-1.00)  - |
| Time in Sweden, years | Age, Gender, Unaccompanied | 1  2  3  4 | - | 1.20 (0.99-1.46)  - | 1.11 (0.92-1.34)  0.92 (0.73-1.17)  - | 0.90 (0.75-1.08)  0.75 (0.59-0.95)  0.81 (0.64-1.03)  - |
| Parental education  Low  Ref: High | Region | 1  2  3  4 | - | 1.68 (0.67-4.23)  - | 2.51 (0.86-7.31)  1.49 (0.41-5.47)  - | 1.50 (0.66-3.44)  0.89 (0.30-2.71)  0.60 (0.18-2.06)  - |
| Parental education  Medium  Ref: High | Region | 1  2  3  4 | - | 1.09 (0.34-3.54)  - | 2.58 (0.78-8.49)  2.36 (0.50-11.00)  - | 1.34 (0.50-3.60)  1.23 (0.31-4.94)  0.52 (0.13-2.12)  - |
| Parental education  Low  Ref: Medium | Region | 1  2  3  4 | - | 1.54 (0.51-4.69)  - | 0.97 (0.35-2.72)  0.63 (0.16-2.47)  - | 1.12 (0.42-2.99)  0.73 (0.19-2.75)  1.15 (0.33-4.06)  - |
| ARQ Self | Unaccompanied, Time in Sweden, Exposure to violence, Number of diagnoses | 1  2  3  4 | - | 0.72 (0.44-1.17)  - | 0.91 (0.62-1.33)  1.27 (0.75-2.14)  - | 1.07 (0.77-1.52)  1.50 (0.90-2.50)  1.19 (0.76-1.84)  - |
| ARQ Family | Unaccompanied, Exposure to violence, Number of diagnoses | 1  2  3  4 | - | 1.32 (0.78-2.22)  - | 0.98 (0.66-1.45)  0.74 (0.44-1.26)  - | 0.80 (0.58-1.11)  0.61 (0.37-1.01)  0.82 (0.54-1.24)  - |
| ARQ Peers | Time in Sweden, Exposure to violence, Number of diagnoses | 1  2  3  4 | - | 0.66 (0.45-0.95)  - | 1.03 (0.74-1.43)  1.57 (1.04-2.36)  - | 0.85 (0.64-1.12)  1.29 (0.90-1.87)  0.83 (0.57-1.19)  - |
| ARQ School | Time in Sweden | 1  2  3  4 | - | 1.04 (0.80-1.35)  - | 1.04 (0.79-1.36)  1.00 (0.71-1.40)  - | 0.86 (0.68-1.08)  0.82 (0.60-1.13)  0.83 (0.60-1.14)  - |
| ARQ Society | Unaccompanied, Time in Sweden, Exposure to violence, Number of diagnoses | 1  2  3  4 | - | 0.90 (0.59-1.37)  - | 1.12 (0.77-1.62)  1.25 (0.80-1.95)  - | 0.73 (0.54-1.00)  0.82 (0.54-1.24)  0.66 (0.44-0.97)  - |
| Exposure to number of different types of violence | Age, Gender, Unaccompanied, Region, Time in Sweden | 1  2  3  4 | - | 1.25 (1.15-1.36)  - | 1.08 (1.01-1.16)  0.87 (0.79-0.95)  - | 1.09 (1.02-1.17)  0.88 (0.80-0.96)  1.01 (0.93-1.10)  - |
| Exposure to sexual violence | Age, Gender, Time in Sweden, Unaccompanied | 1  2  3  4 | - | 5.40 (2.16-13.50)  - | 1.38 (0.51-3.78)  0.26 (0.08-0.82)  - | 2.71 (1.19-6.19)  0.50 (0.18-1.39)  1.96 (0.65-5.94)  - |
| Exposure to child maltreatment | Age, Region, Time in Sweden | 1  2  3  4 | - | 8.12 (2.97-22.20) | 1.66 (0.70-3.94)  0.20 (0.06-0.68)  - | 1.47 (0.69-3.11)  0.18 (0.06-0.58)  0.89 (0.31-2.51)  - |
| Number of diagnoses | Unaccompanied, Time in Sweden, Exposure to violence | 1  2  3  4 | - | 7.55 (3.82-14.90)  - | 4.09 (2.12-7.89)  0.54 (0.35-0.83)  - | 3.58 (1.88-6.84)  0.48 (0.31-0.74)  0.88 (0.55-1.40)  - |
| Internalizing diagnosis | Age, Gender, Time in Sweden, Unaccompanied, Exposure to violence | 1  2  3  4 | - | 37.40 (10.60-132)  - | 7.72 (2.35-25.40)  0.21 (0.06-0.69)  - | 7.72 (2.54-23.50)  0.21 (0.07-0.63)  1.00 (0.33-3.00)  - |
| Sought treatment | Gender, Parental education, Time in Sweden, Unaccompanied | 1  2  3  4 | - | 7.34 (2.75-19.60)  - | 3.23 (1.20-8.69)  0.44 (0.14-1.44)  - | 1.50 (0.56-3.99)  0.20 (0.07-0.64)  0.46 (0.14-1.51)  - |

Note: Data in Class 2, 3, and 4 columns are expressed as odds ratios or adjusted odds ratios with 95% confidence intervals in parantheses. ARQ = Adolescent Resilience Questionnaire

**Table S8: Additional regression analysis with varying references classes.**

| **Independent variable** | **Confounders** | **Reference class** | **Class 1 Good mental health (n = 150)** | **Class 2 Severe mental distress (n = 35)** |
| --- | --- | --- | --- | --- |
| Age | Region of origin | 2  4 | 0.91 (0.81-1.01)  0.92 (0.83-1.02) |  |
| Asylum status:  Awaiting decision  Ref: Received asylum | Unaccompanied, Region of origin, Time in Sweden, Parental education | 4 |  | 7.98 (1.66-38.40) |
| Exposure to number of different types of violence | Age, Gender, Unaccompanied, Region of origin, Time in Sweden | 2  3  4 | 0.80 (0.74-0.87)  0.92 (0.86-0.99)  0.92 (0.86-0.98) | -  1.15 (1.05-1.27)  1.14 (1.04-1.25) |
| Number of diagnoses | Unaccompanied, Time in Sweden, Exposure to violence | 2  3  4 | 0.13 (0.07-0.26)  0.25 (0.13-0.47)  0.28 (0.15-0.53) | -  1.85 (1.20-2.84)  2.11 (1.36-3.26) |
| Exposure to child maltreatment | Age, Region of origin, Time in Sweden | 3  4 |  | 4.90 (1.46-16.40)  5.54 (1.72-17.80) |
| Exposure to sexual victimization | Age, Gender, Time in Sweden, Unaccompanied | 3 |  | 3.90 (1.21-12.60) |
| Sought treatment | Gender, Parental education, Time in Sweden, Unaccompanied | 4 |  | 4.90 (1.56-15.30) |

**References**

1. Swedish Standard Classification of Education (2020).

2. Haahr-Pedersen I, Ershadi AE, Hyland P, et al. Polyvictimization and psychopathology among children and adolescents: A systematic review of studies using the Juvenile Victimization Questionnaire. *Child Abuse Negl*. Sep 2020;107:104589. doi:10.1016/j.chiabu.2020.104589

3. Meinck F, Neelakantan L, Steele B, et al. Measuring Violence Against Children: A COSMIN Systematic Review of the Psychometric Properties of Child and Adolescent Self-Report Measures. *Trauma Violence Abus*. Jul 2023;24(3):1832-1847. doi:10.1177/15248380221082152

4. Mathews B, Pacella R, Dunne MP, Simunovic M, Marston C. Improving measurement of child abuse and neglect: A systematic review and analysis of national prevalence studies. *PLoS One*. 2020;15(1):e0227884. doi:10.1371/journal.pone.0227884

5. Pinto-Cortez C, Gutiérrez-Echegoyen P, Henríquez D. Child Victimization and Polyvictimization Among Young Adults in Northern Chile. *J Interpers Violence*. Mar 2021;36(5-6):2008-2030. doi:10.1177/0886260518759058

6. Emmerich OLM, Wagner B, Heinrichs N, van Noort BM. Lifetime victimization experiences, depressiveness, suicidality, and feelings of loneliness in youth in care. *Child Abuse Negl*. Aug 2024;154:106870. doi:10.1016/j.chiabu.2024.106870

7. Müller LRF, Gossmann K, Schmid RF, Rosner R, Unterhitzenberger J. A pilot study on ecological momentary assessment in asylum-seeking children and adolescents resettled to Germany: Investigating compliance, post-migration factors, and the relation between daily mood, sleep patterns, and mental health. *PLoS One*. 2021;16(2):e0246069. doi:10.1371/journal.pone.0246069

8. Sachser C, Berliner L, Holt T, et al. International development and psychometric properties of the Child and Adolescent Trauma Screen (CATS). *J Affect Disord*. Mar 1 2017;210:189-195. doi:10.1016/j.jad.2016.12.040

9. Nesterko Y, Jäckle D, Friedrich M, Holzapfel L, Glaesmer H. Prevalence of post-traumatic stress disorder, depression and somatisation in recently arrived refugees in Germany: an epidemiological study. *Epidemiol Psychiatr Sci*. 2020;29:e40. e40. doi:10.1017/S2045796019000325

10. Aldabbour B, Abuabada A, Lahlouh A, et al. Psychological impacts of the Gaza war on Palestinian young adults: a cross-sectional study of depression, anxiety, stress, and PTSD symptoms. *BMC Psychology*. 2024/11/26 2024;12(1):696. doi:10.1186/s40359-024-02188-5

11. Brooks MA, Dasgupta A, Khadra M, Bawaneh A, Kaushal N, El-Bassel N. Suicidal behaviors among refugee women in Jordan: post-traumatic stress disorder, social support and post-displacement stressors. *BMC Public Health*. 2024/09/30 2024;24(1):2677. doi:10.1186/s12889-024-20128-1

12. Tinghög P, Malm A, Arwidson C, Sigvardsdotter E, Lundin A, Saboonchi F. Prevalence of mental ill health, traumas and postmigration stress among refugees from Syria resettled in Sweden after 2011: a population-based survey. *BMJ Open*. 2017;7(12):e018899. doi:10.1136/bmjopen-2017-018899

13. Sander R, Laugesen H, Skammeritz S, Mortensen EL, Carlsson J. Interpreter-mediated psychotherapy with trauma-affected refugees – A retrospective cohort study. *Psychiatry Res*. 2019/01/01/ 2019;271:684-692. doi:10.1016/j.psychres.2018.12.058

14. Ghandour R, Hammoudeh W, Stigum H, Giacaman R, Fjeld H, Holmboe-Ottesen G. The hidden burden of dysmenorrhea among adolescent girls in Palestine refugee camps: a focus on well-being and academic performance. *BMC Public Health*. 2024/03/06 2024;24(1):726. doi:10.1186/s12889-024-18219-0

15. Nilsson D, Svedin CG, Hall F, Kazemi E, Dahlström Ö. Psychometric properties of the Adolescent Resilience Questionnaire (ARQ) in a sample of Swedish adolescents. *BMC Psychiatry*. 2022;22(1)doi:10.1186/s12888-022-04099-4

16. Gartland D, Bond L, Olsson CA, Buzwell S, Sawyer SM. Development of a multi-dimensional measure of resilience in adolescents: the Adolescent Resilience Questionnaire. *BMC Med Res Methodol*. 2011;11(1):134. doi:10.1186/1471-2288-11-134

17. Högberg C, Billstedt E, Björck C, et al. Diagnostic validity of the MINI-KID disorder classifications in specialized child and adolescent psychiatric outpatient clinics in Sweden. *BMC Psychiatry*. 2019/05/09 2019;19(1):142. doi:10.1186/s12888-019-2121-8

18. Kyrillos V, Bosqui T, Moghames P, et al. The culturally and contextually sensitive assessment of mental health using a structured diagnostic interview (MINI Kid) for Syrian refugee children and adolescents in Lebanon: Challenges and solutions. *Transcult Psychiatry*. 2023;60(1):125-141. doi:10.1177/13634615221105114

19. Hocking DC, Mancuso SG, Sundram S. Development and validation of a mental health screening tool for asylum-seekers and refugees: the STAR-MH. *BMC Psychiatry*. 2018/03/16 2018;18(1):69. doi:10.1186/s12888-018-1660-8

20. Bogic M, Njoku A, Priebe S. Long-term mental health of war-refugees: a systematic literature review. *BMC Int Health Hum Rights*. 2015;15(1)doi:10.1186/s12914-015-0064-9
